# Developing and feasibility testing of data collection methods for an economic evaluation of a supported selfmanagement programme for adults with a learning disability and type 2 diabetes

**DOI:** 10.1186/s40814-018-0266-8

**Published:** 2018-04-23

**Authors:** John L. O’Dwyer, Amy M. Russell, Louise D. Bryant, Rebecca E. A. Walwyn, Alexandra M. Wright-Hughes, Elizabeth H. Graham, Judy M. Wright, Shaista Meer, Jacqueline Birtwistle, Amanda J. Farrin, Allan O. House, Claire T. Hulme

**Affiliations:** 1 Academic Unit of Health Economics, Leeds Institute of Health Sciences, University of Leeds, Worsley Building, Leeds, LS2 9NL UK; 2 Leeds Institute of Clinical Trials Research, University of Leeds, Worsley Building, Leeds, LS2 9NL UK

**Keywords:** Type 2 diabetes, Supported self-management, Learning disability, Economic evaluation, EQ-5D

## Abstract

**Background:**

The challenges of conducting research with hard to reach vulnerable groups are particularly pertinent for people with learning disabilities. Data collection methods for previous cost and cost-effectiveness analyses of health and social care interventions targeting people with learning disabilities have relied on health care/health insurance records or data collection forms completed by the service provider rather than by people with learning disabilities themselves. This paper reports on the development and testing of data collection methods for an economic evaluation within a randomised controlled trial (RCT) for a supported self-management programme for people with mild/moderate learning disabilities and type 2 diabetes.

**Methods:**

A case finding study was conducted to identify types of health and social care use and data collection methods employed in previous studies with this population. Based on this evidence, resource use questionnaires for completion by GP staff and interviewer-administered participant questionnaires (covering a wider cost perspective and health-related quality of life) were tested within a feasibility RCT. Interviewer-administered questionnaires included the EQ-5D-3L (the NICE recommended measure for use in economic evaluation). Participants were adults > 18 years with a mild or moderate learning disability and type 2 diabetes, with mental capacity to give consent to research participation.

**Results:**

Data collection for questionnaires completed by GP staff requesting data for the last 12 months proved time intensive and difficult. Whilst 82.3% (121/147) of questionnaires were returned, up to 17% of service use items were recorded as unknown. Subsequently, a shorter recall period (4 months) led to a higher return rate but with a higher rate of missing data. Missing data for interviewer-administered participant questionnaires was > 8% but the interviewers reported difficulty with participant recall. Almost 60% (48/80) of participants had difficulty completing the EQ-5D-3L.

**Conclusions:**

Further investigation as to how service use can be recorded is recommended. Concerns about the reliability of identifying service use data directly from participants with a learning disability due to challenges in completion, specifically around recall, remain. The degree of difficulty to complete EQ-5D-3L indicates concerns regarding the appropriateness of using this measure in its current form in research with this population.

**Trial registration:**

Current Controlled Trials ISRCTN41897033 (registered 21 January 2013).

**Electronic supplementary material:**

The online version of this article (10.1186/s40814-018-0266-8) contains supplementary material, which is available to authorized users.

## Background

It is estimated that there are around 1.2 million people in the UK with a mild or moderate learning disability [1]. Learning disability can be defined as the presence of a significantly reduced ability to understand new or complex information and to learn new skills combined with a reduced ability to cope independently [2]. As a population, people with a learning disability are more likely to be in poorer health and to die earlier than those without a learning disability [3, 4]. Disparities in health and health outcomes for this population have been the focus of recent NHS policy initiatives, which have placed addressing poor outcomes and health inequalities for people with a learning disability at the heart of their agenda [5]. These disparities can be seen in the higher prevalence of type 2 diabetes in people with a learning disability compared to the general population, 9–11 and 4–5%, respectively [6, 7], and higher rates of hospital admissions due to poorly controlled diabetes [6, 8].

There are a number of possible explanations for high rates of poorly controlled type 2 diabetes in adults with a learning disability including high prevalence of obesity and unhealthy diets, prescription medications that increase risk of obesity, reduced self-management skills and lack of practical support [9–11]. Whilst self-management of type 2 diabetes is encouraged in the general population, its value and cost effectiveness has not been explored in people with a learning disability.

**Fig. 1:**
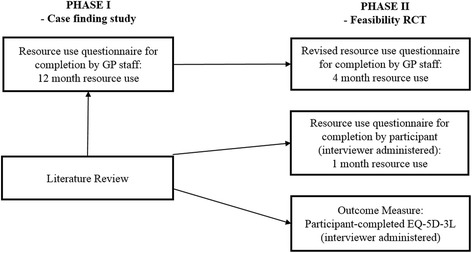
Phase I and phase II data collection development

This paper reports on the development and testing of data collection methods for an economic evaluation within a randomised controlled trial (RCT) for a supported diabetes self-management programme for people with a mild/moderate learning disability. A full description of the study is reported elsewhere [12], but in brief the study sought to explore the feasibility of conducting a definitive phase III randomised controlled trial to evaluate the clinical and cost-effectiveness of supported self-management of type 2 diabetes in adults with a mild or moderate learning disability and consisted of the following: (i) an initial case finding and recruitment study, (ii) development of materials to implement and evaluate an RCT of supported self-management, (iii) feasibility RCT of supported self-management + treatment as usual (SSM) vs. treatment as usual (TAU). As Fig. 1 shows, development and testing of data collection methods for an economic evaluation was undertaken in two phases, during the case finding study (phase I) and during the feasibility randomised controlled trial (RCT) (phase II).

## Phase I—case finding study; methods for economic evaluation

The aim of the phase I case finding study was to identify the different types of health and social care used by individuals with a mild/moderate learning disability and type 2 diabetes. This information would then be used to design a resource use questionnaire for completion by GP staff using the patient’s record for use in an economic evaluation of a supported self-management intervention for this population. In addition, phase I would identify suitable outcome measures for such an economic evaluation.

A literature review was undertaken to inform the choice of outcome measure and the development of the health care resource use questionnaires to be completed by GP staff. These health care resource use questionnaires were to be piloted within the phase I case finding study before further refinement and testing for use in the phase II feasibility RCT.

### Literature review

The aim of the literature review was fourfold: to identify (i) health and social care resources used by people with type 2 diabetes and a learning disability within self-management interventions (including frequency and drivers of costs) and the methods by which that data were collected; (ii) health and social care resources used by the general population of people with type 2 diabetes within self-management interventions (including frequency and drivers of costs); (iii) health and social care resources used in other settings by people with a learning disability and the methods by which that data was collected; (iv) outcome measures used within any of the cost-effectiveness analyses identified.

The search strategies comprised index terms, text words and their synonyms for four concepts: learning disability, self-care interventions, type 2 diabetes and costs (or economic evaluations). Three separate searches were run in EMBASE, MEDLINE and the Cochrane Library between May 2013 and February 2014. Searches used alternative combinations of three of the four concepts in each. The searches were not limited by language or date of publication. The inclusion criteria were as follows: cost-effectiveness studies for healthy lifestyle interventions; cost-effectiveness studies for diabetes self-management; and studies with costs and/or outcomes for people with a learning disability. Papers were excluded in which studies reported in abstract form only, related to self-monitoring or focussed on pharmaceutical treatment for diabetes. Titles and abstracts were reviewed by two people (CH and JOD) and data extracted using a bespoke data extraction template which included the type of intervention, duration of the study, outcome measures used and type of cost data collected.

### Development and administration of resource use questionnaires

Based on information retrieved from the literature review (see Additional file 1), together with input from the wider study team, the health care resource use questionnaires to be completed by GP staff were developed. This questionnaire covered primary and secondary health care use over a 12-month period and included community-based services such as an ophthalmologist, podiatrist and dietician, i.e. services that people with diabetes are often advised to attend.

After consent to contact their GP was obtained from the study participant, the Clinical Trials Research Unit (CTRU) posted the questionnaire to GP practices. A cover letter explaining the study and reimbursement for the time to complete the questionnaire was provided. The CTRU informed the research team if the questionnaire had not been returned after 6 weeks. Reminders to complete questionnaires were by way of a series of emails and unlogged telephone calls by a researcher to the GP practices. For questionnaires still not returned after the 6-week reminder, where feasible, the researcher visited the GP surgery and supported the practice to complete the questionnaire.

The completeness of the data collected in the questionnaires was recorded using descriptive statistics, detailing the number and percentage of questionnaires returned and the number and percentage of missing items within the returned questionnaires.

## Phase I—case finding study; results

The literature review searches identified 1189 unique references.

### Resource use

The first search of evidence of cost-effectiveness analysis of self-management interventions in people with diabetes and a learning disability found no papers that met the inclusion criteria. The second search, studies of cost-effectiveness analysis of self-management interventions in people with diabetes, identified eight studies [13–20] that met the inclusion criteria (see Additional file 2). The selected studies were lifestyle modification programmes and telephone interventions (both automated and non-automated). The final search for studies detailing costs or costs and outcomes of interventions for people with a learning disability, identified six studies [21–26] that met the inclusion criteria (see Additional file 2). Two cost-of-illness studies [22, 24] examined service use and healthcare costs of people with a learning disability, giving an overview of the main drivers in the cost of care for a person with a learning disability, with hospital-based care, GP, nursing care and accommodation being the main drivers of service costs.

Cost data were collected using a variety of methods including interviews with health workers, standardised templates for staff, participant questionnaires and participant medical records. Each diabetes self-management intervention included programme costs with staff (e.g. nurses, counsellors, educators) being the greatest drivers of cost in relation to intervention delivery. Administration costs such as telephone charges, printing charges and translation costs added to the expense of the interventions. In many of the US studies, patient costs were not included or included in a generalised form using insurance information, with just one study [15] including participant out-of-pocket costs such as gym membership, exercise equipment and cost of diet change. From Review (iii), resource use (hospital-based care, GP and nurse care) and accommodation were found to be the main drivers identified in relation to the cost-of-illness studies for people with a learning disability. The UK studies [21, 22, 25, 26] used modified versions of the Client Service Receipt Inventory (CSRI) [27].

Based on the limited data of health and social care use gleaned from the review in phase I of this study, and in consultation with the experts on the study team, a resource use questionnaire to be completed by GP staff was developed using a modified version of the CSRI for distribution to GP practices.

### Outcome measures

In respect of outcomes, the included diabetes interventions used clinical marker data such as HbA1C levels, body mass index (BMI) and risk of cardiovascular disease; in terms of quality of life measures, the majority of the diabetes interventions used quality-adjusted life years (QALYs) [13, 17–20], derived from the EQ-5D-3L or SF-12. In the four studies involving a cohort of people with cognitive or learning disability which measured health outcomes, two studies [23, 25] used activity patterns such as social interactions; another study [26] measured health outcomes using an aggression scale and the QOL-Q; and one cost-effectiveness analysis [21] used EQ-5D-3L to calculate QALYs.

Previous work has suggested that the SF-36 is promising for use with people with a learning disability [28]; the shorter SF-12, used in one of the papers identified [17], uses 12 questions from the SF-36. The EQ-5D-3L and the Health Utilities Index (HUI) have also been shown to be superior compared with other preference-based measures of health for this population [28]. None of the studies identified in Reviews (ii) and (iii) had used the HUI. Based on the frequent use of EQ-5D-3L in the included literature from Review (ii), the EQ-5D-3L was chosen to be assessed for feasibility as an outcome measure for any subsequent RCT within phase II.

#### Data collection experience and missing data results

Between June 2013 and January 2015, a resource use questionnaire to be completed by GP staff for each consented participant was posted to their GP practice for completion using their patient record. The questionnaires asked about the services the participant had used over the previous 12 months. Case finding methods in phase 1 identified 147 eligible and consenting participants, of whom 121 (82.3%) had a resource use questionnaire completed on their behalf by a member of staff at a GP practice or with support from a researcher. Of completed questionnaires, missing data for each item was < 5%; however, items recording service use responses, whilst not missing, were recorded as unknown in up to 17% of items. The data collection process for these questionnaires proved time intensive and difficult. In some instances, researcher telephone reminders to encourage completion exceeded nine or more attempts. Further attempts to contact proved less rewarding with emails being rarely replied to. Often service uses could only be accurately counted by opening every document in the participant’s file to check for referral letters which had been scanned and saved as attachments in a patient record. There were cases where staff at practices refused to complete sections because the process took longer than they had anticipated. Those practices who did not want to complete the questionnaire were offered the option of a research team member visiting the practice to support them in completing the questionnaire; however, this offer was only accepted on three occasions.

#### Resource use

As shown in Table 1, GP and practice nurse visits are the most frequent resources used. Almost 93% of the 121 participants visited the GP at least once in a 12-month period and 90% of participants visited the practice nurse. One in three (34.7%) participants had been to see a podiatrist and over one quarter (27.3%) had been to see an ophthalmologist. Over 21% of participants attended A&E in the 12-month period and 11.6% had at least one inpatient stay. Services such as dietician, diabetic clinic, district nurse, and nephrologist were much less frequently used. Of the 121 questionnaires received, only three participants (2.5%) had been recorded attending a nephrology appointment during a 12-month period.

**Table 1:** Service usage phase I (12 months)

Completed GP questionnaire (*n* = 121)	Yes	No	Unknown	Missing
GP	112 (92.6%)	7 (5.7%)	2 (1.7%)	0 (0.0%)
Practice nurse	109 (90.1%)	10 (8.3%)	1 (0.8%)	1 (0.8%)
District nurse	14 (11.6%)	83 (68.6%)	20 (16.5%)	4 (3.3%)
Diabetic clinic at the hospital	9 (7.4%)	97 (80.2%)	10 (8.3%)	5 (4.1%)
Ophthalmologist	33 (27.3%)	67 (55.4%)	16 (13.2%)	5 (4.1%)
Podiatrist	42 (34.7%)	61 (50.4%)	15 (12.4%)	3 (2.5%)
Dietician	12 (9.9%)	86 (71.1%)	20 (16.5%)	3 (2.5%)
Nephrologist	3 (2.5%)	99 (81.8%)	13 (10.7%)	6 (5.0%)
Inpatient stays	14 (11.6%)	91 (75.2%)	11 (9.1%)	5 (4.1%)
A&E	26 (21.5%)	75 (62.0%)	15 (12.4%)	5 (4.1%)

The questionnaire also included a space to record prescribed diabetes medications. Of the 121 completed questionnaires, diabetes medication information was supplied for 61 participants (50.4%). Additional file 3: Table S3 shows the medication information supplied. Metformin was the most frequently recorded drug, being received by over 81% of participants (50/61) for whom completed questionnaires were returned.

## Phase II—feasibility RCT; methods

In addition to the resource use questionnaire to be completed by GP staff developed for the phase I case finding study, phase II questionnaires for completion by the study participants (through interviewer-administration) were developed based on the findings from the literature review (phase I) and input from the study team. A secondary aim of phase II was to test the data collection questionnaires for use in a subsequent definitive RCT. This included an analysis of the acceptability of interviewer-administered participant questionnaires and participant-reported outcomes from interviews at baseline and 6-month follow-up, and an exploratory analysis of data collected from GP records using a revised version of the phase I questionnaire to be completed by GP staff in order to identify the main drivers of health and social care cost within the intervention and treatment-as-usual (TAU) groups.

The resource use questionnaire to be completed by participants took a wider, societal perspective including questions about health care use resource use, employment status, sick leave, their living situation and out-of-pocket expenditures incurred as a result of health care admissions or appointments. Questions were written in plain English and were phrased in a conversational tone. The interviewers recorded post-interview journals reporting on their perceptions of the ease of completion. This gave a more in-depth understanding of the challenges for participants in responding to the questions, and the influence of supporters on participant responses. The questionnaires were analysed using descriptive statistics to assess the level of completeness of data, categorised into resource use, employment and accommodation.

In addition to the questionnaire for completion by participants, a revised resource use questionnaire was again sent to GP practices to complete using the patient’s record. As concerns were voiced by staff at the GP practices and study researchers over the time-consuming and problematic collection process in phase I, for phase II, there was a change of approach to the collection of health care resource use data. GP practices were asked for participants’ resource use relating to a shorter duration than in phase I (4 months rather than 12 months). The completion rate improved for phase II, GP practices were contacted regarding completion after 6 weeks had passed on just two occasions.

Unit costs were assigned to the data in order to estimate the main drivers of health and social care costs over a 4-month period for the sample. Unit costs were obtained from national sources including the Personal Social Services Research Unit (PSSRU) Costs of Health and Social Care [29], NHS reference cost database [30] and the British National Formulary (BNF) [31]. The cost analysis was based on complete cases only (i.e. questionnaires which had no missing or unknown data).

Based on findings from the literature review in phase I, the EQ-5D-3L was chosen to be assessed as part of the phase II feasibility study [32, 33] and included as part of the interviewer-administered questionnaire. No changes were made to the text of the EQ-5D-3L; however, each EQ-5D-3L domain, and associated levels, were printed on a separate A4 laminated sheet as an interview aid. The visual analogue scale (VAS) element of the EQ-5D-3L was not included as the service user involvement group who work with people with learning disabilities felt it would be too difficult for participants to understand. This view accords with another study [34].

## Phase II—feasibility RCT; results

### Participant-reported resource use and wider societal perspective

Participants were interviewed at baseline and at 6-month follow-up. Interviews were undertaken between September 2014 and September 2015. At baseline, 82 participants were interviewed, 40 males and 42 females and the mean age was 56.4 years; 77 of these participants were interviewed for a second time at follow-up. A full description of the complex recruitment process is reported elsewhere [12].

#### Accommodation

We asked participants about their living arrangements. In relation to accommodation type at baseline, 57.3% of participants (47/82) lived in domestic housing; 15.9% (13/82) lived in sheltered housing; 18.3% (15/82) lived in a shared supported house. At follow-up, 50.6% of participants (39/77) lived in domestic housing, 16.9% (13/77) lived in sheltered housing and 26% (20/77) lived in a shared supported house. There was only one missing observation across the sample for this question; however, interviewer notes highlight the difficulty in being able to categorise their residence; for the 77 participants interviewed at follow-up, over 40% changed their answer from the one they gave at baseline (see Additional file 3: Table S4).

#### Employment

As shown in Table 2, over half of the participants (baseline 54.9% (45/82); follow-up 51.9% (40/77)) were ‘At home, unable to work’. At each time point, almost one fifth of participants (baseline 17.1% (14/82); follow-up 19.5% (15/77)) classed themselves as ‘Retired’. At baseline, 14.6% of participants (12/82) were in employment, and at follow-up, 10.4% (8/77) were in employment. There was missing data from just one participant; however, interviewers recorded 31 participants (37.8%) having difficulty categorising their employment status at baseline. The employment status changed for 39 of the 77 participants (50.6%) who completed questionnaires from baseline to follow-up, with 19 of these (24.7%) appearing to move in both directions between ‘Retired’ and ‘At home and unable to work’.

**Table 2:** Baseline service use (1 month)

Service (*n* = 82)	Yes	No	Don’t know	Missing	Mean times	Min	Max
Saw GP at the surgery	40 (48.8%)	40 (48.8%)	2 (2.4%)	0 (0.0%)	1.35	1	3
Saw GP at home	1 (1.2%)	79 (96.3%)	2 (2.4%)	0 (0.0%)	3.00	3	3
Saw a nurse	4 (4.9%)	75 (91.5%)	3 (3.7%)	0 (0.0%)	2.25	1	3
Phoned a nurse for advice	32 (39.0%)	48 (58.5%)	2 (2.4%)	0 (0.0%)	1.55	1	4
Got a repeat prescription	1 (1.2%)	79 (96.3%)	2 (2.4%)	0 (0.0%)	1.00	1	1
Got meals on wheels	51 (62.2%)	25 (30.5%)	6 (7.3%)	0 (0.0%)	1.32	1	4
Home help came around	0 (0.0%)	80 (97.6%)	2 (2.4%)	0 (0.0%)	0.00	0	0
Saw social worker	14 (17.1%)	67 (81.7%)	1 (1.2%)	0 (0.0%)	7.45	1	28
Been to A&E	4 (4.9%)	77 (93.9%)	0 (0.0%)	1 (1.2%)	1.00	1	1
Stayed in hosp. overnight	2 (2.4%)	79 (96.3%)	0 (0.0%)	1 (1.2%)	1.5	1	2
Outpatient	31 (37.8%)	50 (61.0%)	1 (1.2%)	0 (0.0%)	1.452	1	4

Participants were asked if they had been off work due to illness in the last 4 weeks. Three participants (3.7%) indicated they had at baseline, and just one participant indicated sick leave at follow-up. All sick leave recorded was between 1 and 2 days.

#### Participant-reported service use

Participants were asked to recall the health and social care services they had used in the last 4 weeks. The results are shown in Tables 3 and 4.

**Table 3:** Current employment situation

Employment status	Baseline	Follow-up
Working full time	3 (3.7%)	4 (5.2%)
Working part time	9 (11.0%)	4 (5.2%)
Unemployed and looking for work	0 (0.00%)	1 (1.3%)
At home/unemployed and not looking for work	1 (1.2%)	0 (0.0%)
At home, unable to work	45 (54.9%)	40 (52.0%)
Volunteer	7 (8.5%)	10 (13.0%)
Student	1 (1.2%)	0 (0.0%)
Retired	14 (17.1%)	15 (19.5%)
Other	1 (1.2%)	3 (3.9%)
Missing	1 (1.2%)	0 (0.0%)
Total	82 (100.0%)	77 (100.0%)

**Table 4:** Follow-up service use (1 month)

Service (*n* = 77)	Yes	No	Don’t know	Missing	Mean times	Min	Max
Saw GP at the surgery	26 (33.8%)	47 (61.0%)	4 (5.2%)	0 (0.0%)	1.44	1	5
Saw GP at home	3 (3.9%)	74 (96.1%)	0 (0.0%)	0 (0.0%)	2.00	1	3
Saw a nurse	13 (16.9%)	64 (83.1%)	0 (0.0%)	0 (0.0%)	1.92	1	5
Phoned a nurse for advice	23 (29.9%)	50 (64.9%)	3 (3.9%)	1 (1.3%)	1.36	1	6
Got a repeat prescription	1 (1.3%)	73 (94.8%)	1 (1.3%)	2 (2.6%)	3.00	3	3
Got meals on wheels	73 (94.8%)	4 (5.2%)	0 (0.0%)	0 (0.0%)	1.00	1	1
Home help came around	0 (0.0%)	77 (100.0%)	0 (0.0%)	0 (0.0%)	0.00	0	0
Saw social worker	14 (18.2%)	61 (79.2%)	1 (1.3%)	1 (1.3%)	8.75	1	28
Been to A&E	4 (5.2%)	72 (93.5%)	0 (0.0%)	1 (1.3%)	2.00	1	5
Stayed in hosp. overnight	3 (3.9%)	73 (94.8%)	0 (0.0%)	1 (1.3%)	4.00	1	10
Outpatient	16 (20.8%)	60 (77.9%)	1 (1.3%)	0 (0.0%)	1.40	1	4

Very little missing data (< 8%) was reported at baseline and follow-up; however, the interviewers did report a number of respondents having difficulty recalling their health and social care use (62% at baseline and 41% at follow-up). The recall time posed particular problems with supporter clarification required:

- *“*Had to explore if the visit had been in the last four weeks, since participant also mentioned two visits before Christmas.” (#75)

When asked about hospitals stays, some participants would report stays which were found to be from a long time ago despite the researcher defining the timeframe we were interested in. Responses to questions about changes in medication were complicated to interpret as people often did not know what they were taking or only knew medication by shape and/or size of tablet:

- “Person reported hospital stay that happened a long time ago…..Participant knew she took medication and told researcher what tablet was like. Initially suggested that medication had changed but when researcher enquired further it seemed that it had not.” (#81)

Paid support workers often had files for a person and could look up hospital visits and confirm or deny self-reported information. Some participants knew they had made some use of the GP but not the number of times; others may have answered positively to hospital use but have been thinking of visits outside the time frame:

- “Could recall services used but did not know how many times. Participant said had been to hospital in the last six months but on checking with supporter, she had not.” (#344)

#### Self-reported medication

At the follow-up interview, participants were asked if their medication had changed over the past 6 months. Just seven participants (9.1%) indicated it had; four participants (5.2%) indicated a change in frequency, and three participants (3.9%) indicated the type of medication had changed. This data could not be verified.

### GP practice-reported resource use

In order to identify the main drivers of health and social care cost over a 4-month period, we assigned unit costs to each service use (Table 4). Data was not available for the length of stay in hospital; therefore, each recorded inpatient stay was assumed to be a short stay. The cost analysis was based on complete cases only (i.e. questionnaires which had no missing or unknown data), this reduced our sample from 70 (85.4% of recruited participants) to 55 (67.1% of recruited participants).

As shown in Table 5 below, GP and practice nurse visits were the most frequent resources used. Over 77% of the 70 participants visited the GP at least once in a 4-month period and 60% of participants visited the practice nurse. Again, visits by participants to see a podiatrist were close to 30% with 10% having been to see an ophthalmologist. Over 14% of participants attended A&E in the 4-month period and 10% had at least one inpatient stay. Services such as dietician, diabetic clinic, district nurse, and nephrologist were much less frequently used. From 70 questionnaires received, only one participant had recorded attending a nephrology appointment during a 4-month period.

**Table 5:** Service usage phase II: (4-month period)

Completed GP questionnaires: (*n* = 70)	Yes	No	Unknown	Missing
GP	54 (77.1%)	15 (21.4%)	0 (0.0%)	1 (1.4%)
Practice nurse	42 (60.0%)	25 (35.7%)	0 (0.0%)	3 (4.3%)
District nurse	4 (5.7%)	58 (82.9%)	4 (5.7%)	4 (5.7%)
Diabetic clinic at the hospital	3 (4.3%)	60 (85.7%)	1 (1.4%)	6 (8.6%)
Ophthalmologist	7 (10.0%)	55 (78.6%)	2 (2.9%)	6 (8.6%)
Podiatrist	20 (28.6%)	42 (60.0%)	3 (4.3%)	5 (7.1%)
Dietician	3 (4.3%)	60 (85.7%)	1 (1.4%)	6 (8.6%)
Nephrologist	1 (1.4%)	64 (91.4%)	0 (0.0%)	5 (7.1%)
Diabetes educational course	0 (0.0%)	61 (87.1%)	3 (4.3%)	6 (8.6%)
Chronic illness course	2 (2.9%)	59 (84.3%)	3 (4.3%)	6 (8.6%)
Inpatient stays	7 (10.0%)	59 (84.3%)	1 (1.4%)	3 (4.3%)
A&E	10 (14.3%)	55 (78.6%)	1 (1.4%)	4 (5.7%)

An exploratory analysis was undertaken of the main drivers of cost by assigning unit costs to the data recorded on the questionnaires completed by GP staff, excluding medications (Table 6).

**Table 6:** Service use: costs (assuming inpatient short stays) for complete cases for 4 months

Variable	*n*	Mean no. of visits	Mean cost (£)	Min cost (£)	Max cost (£)
GP	55	3.13	143.85	0.00	1058.00
Practice nurse	55	1.18	16.18	0.00	95.83
District nurse	55	0.05	1.40	0.00	51.48
Diabetic clinic at the hospital	55	0.04	3.82	0.00	105.00
Ophthalmologist	55	0.13	9.04	0.00	142.00
Podiatrist	55	0.36	11.64	0.00	64.00
Dietician	55	0.05	2.02	0.00	37.00
Nephrologist	55	0.00	0.00	0.00	0.00
Diabetes educational course	55	0.00	0.00	0.00	0.00
Chronic illness course	55	0.04	2.36	0.00	65.00
Inpatient stays	55	0.13	77.76	0.00	1222.00
A&E	55	0.16	29.45	0.00	360.00
Total	55	5.27	321.89	0.00	1947.00

#### Medications

The questionnaire completed by GP staff also included a space to record prescribed diabetes medications. Additional file 3: Table S5 highlights the diabetes medications prescribed to the participants and the cost for 4 months.

Additional file 3: Table S6 highlights the medication use of the 55 participants with complete information. Metformin, again, remains the most frequently used drug, being received by the majority of participants.

### Outcomes

In order to calculate QALYs, the feasibility of using the EQ-5D-3L in a larger trial was explored. Table 7 provides a summary of participant-reported EQ-5D-3L over time.

**Table 7:** EQ-5D-3L by time point

EQ-5D-3L	*n*	Mean	SD	Min	Lower quartile	Median	Upper quartile	Max
Baseline	80	0.6668	0.2875	0.239	0.603	0.725	0.848	1
Follow-up	76	0.6641	0.3458	0.239	0.431	0.788	1	1

The EQ-5D was incomplete for two participants at baseline and one out of 77 participants with a follow-up visit. The average absolute difference between baseline and follow-up was 0.0042 (SD 0.2893). Despite few missing data, interviewers reported that 60% of participants (48/80) at baseline and 53.9% of participants (41/76) at follow-up had some level of difficulty completing the measure. The most important contextual area was the role played by the supporter in the interview process. Although 25% of participants (20/80) did not have a supporter present, for those with a supporter present interviewers recorded supporter intervention on occasion (for 11% of participants at baseline and 14.5% at follow-up) providing explanation of the questions when required, and sometimes when not. Supporters contextualised the question for the respondent, answering questions on the respondent’s behalf and sometimes succeeding in persuading respondents to change answers to match their own perceptions of the respondent’s health state. In the case of the latter, it was clear that often the supporter did not agree with the level chosen by the participant. On occasion, the supporter being present was enough to influence the participant. Researchers recorded the following observation:

- “Participants answers were contradicted by the supporter at first. This led to participant asking first what supporter would say before giving own answer. I tried to capture participants own answers.” (#91)

The linguistic challenges and the role played by the supporter were not mutually exclusive and often overlapped. At baseline, 28.8% of participants (23/80) were assisted by a supporter; at follow-up, this figure decreased to 21.1% (16/76).

## Discussion

Based on evidence from the literature and expert advice from the research team, we developed two data collection instruments relating to participants’ health and social care use, assessing two methods of data collection. Resource use questionnaires (covering participants’ health and social care) completed by GP staff and interviewer-administered participant questionnaires (covering a wider cost perspective and health-related quality of life using the EQ-5D-3L).

The analysis of the results from the phase I resource use questionnaire completed by GP staff highlights the high use of primary care services such as GP and nurse care in comparison with hospital-based care. Whilst 82.3% of the questionnaires were returned and with few missing items (< 5% missing), there was however a high level of service use recorded as unknown (which could therefore be considered to be missing data). This highlights the challenge of data collection in primary care. Completion of the resource use questionnaires by GP staff was time consuming and problematic in respect of the intensity of follow-up reminders required to achieve the return rate of over 82% (including researchers helping with completion in the GP surgeries). Potential reasons for the time-consuming nature of the questionnaires and the missing data was the 12-month time period over which resource usage was requested and the GP practice inability or unwillingness to complete the task.

In phase II, the time period over which resource usage was collected from GP records was decreased to 4 months, to ease completion. When unit costs were applied, the main drivers of cost were GP care and hospital-based care (A&E and inpatient stays). The types of services used and the main drivers of cost are similar to findings from the literature review [19–22, 25, 26]. The time spent in hospital was not recorded; therefore, each inpatient stay is assumed to be a short stay which may underestimate the cost of this resource use. In addition, it was not possible to differentiate between face-to-face and telephone contacts from the patient’s records; face-to-face contact was assumed which may have overestimated the time and cost.

The intensity of reminders and GP visits to assist completion in case finding was not replicated in the feasibility RCT, and in fact, the return rate increased in the second phase of the study. It is not possible to say whether this was because the surgeries became more adept at completion over time or whether it was because the questionnaires were asking about a shorter timespan in the RCT. There were less data reported as ‘unknown’; however, more data was reported as missing. Data on services provided outside the GP surgery team, some of which are part of the Diabetes Annual Care Review, e.g. podiatrist, which were recorded as unknown and missing remained relatively high. If GP records were to be used in future studies with this population, methods to retrieve data on community service use would also need to be factored into the design. The only service not recorded as being used by any of the participants was the diabetes educational course despite being part of recommended first-line treatment of type 2 diabetes in adults [35]. Further investigation of how these community-based services may be captured is required.

Despite the difficulty in collecting data directly from GP records, it appears to be a more reliable method than collecting the information from participants themselves. Participants were asked to recall their recent health care use at baseline and follow-up. Interviewers recorded a high occurrence of difficulty with this question (61% at baseline). Rephrasing of questions (e.g. 4 weeks/1 month) and supporter clarification was required. Such a high degree of difficulty with recall indicates concerns about collecting resource use data directly from participants. Interviewers classified accommodation and employment subjectively and changed their interpretation of the response (or it was a different interviewer). A finding would be that these categories need to be better defined for interviewers.

The solution as to how to collect resource use data for this population could lie in the use of electronic records, such as Patient Level Information and Costing System (PLICS), as coverage in the UK increases. Providing a central data team with specific data extraction queries could make the data collection process more efficient. These methods bring their own challenges, not least in how to collect out-of-pocket costs for lifestyle change interventions (e.g. cost of changes in diet and exercise) and the costs associated with self-management itself. Accommodation and employment status data may also not be included within GP records.

There was almost no missing EQ-5D-3L data reported with only two participants not completing this part of the questionnaire at baseline and one not completing at follow-up. This was most likely because it was interviewer-administered. Despite high completion rates, participants still experienced difficulty answering, with up to 60% being considered to have some level of difficulty with the EQ-5D-3L questions and requiring assistance from the researcher or supporter. The main difficulty experienced was with wording and understanding terms in the questions; rephrasing and explanations of terms helped with completion; however, this is discouraged by the creators of the EQ-5D as it invalidates the measure. Supporters intervened often, sometimes correcting participant’s answers, even if the participant did not appear to have trouble with the question. Participants on occasion disagreed with supporters, placing a different value on their own health.

## Conclusion

Data collection methods for a future economic evaluation were developed, and their feasibility was tested during this study. In the testing phase (feasibility RCT), there was a higher return rate of the resource use questionnaire from GP practices than had been seen in the development phase (phase I). There were relatively high rates of ‘unknown’ answers for community-provided care than for care provided within the GP surgery or by the GP team; therefore, a requirement for further investigation as to how community team service use can be recorded remains, as does sustaining the high return rate from GP practices.

In respect of the participant-completed resource use questionnaires, there were challenges in completion, specifically around the time particular events happened and recall more generally. Difficulty with recall is common in people who have a learning disability [36], and there are concerns about the reliability of identifying service use data for use in economic evaluations directly from the participants with a learning disability themselves.

In respect of assessment of quality of life, although rephrasing and provision of explanations facilitated completion of EQ-5D-3L, a substantial proportion of participants had difficulty in understanding the questions. The high degree of difficulty experienced indicates concerns regarding the appropriateness of using this measure in its current form in research with this population. Further research into the language, format and process of completion of the EQ-5D-3L for participants with a learning disability is recommended.

## Additional files

Additional file 1: **Table S1.** Cost effectiveness analyses of self-management interventions in people with diabetes. (DOCX 28 kb)

Additional file 2: **Table S2.** Cost effectiveness analysis of interventions for people with learning disabilities. (DOCX 22 kb)

Additional file 3: Other Tables. (DOCX 16 kb)

## Abbreviations

BMI: Body mass index
BNF: British National Formulary
CSRI: Client Service Receipt Inventory
CTRU: Clinical Trials Research Unit
EQ-5D-3L: EuroQol five-dimension (three-level) generic health outcome measure
GP: General practitioner
HUI: Health Utilities Index
PSSRU: Personal Social Services Unit
QALY: Quality-adjusted life year
RCT: Randomised controlled trial
SF-12: 12-Item Short Form Health Survey
SSM: Supported self-management
TAU: Treatment as usual
VAS: Visual analogue scale
