# Supplementary material for: Developing and feasibility testing of data collection methods for an economic evaluation of a supported selfmanagement programme for adults with a learning disability and type 2 diabetes
Source: Pilot Feasibility Stud. 2018 Apr 23;4:80. doi: 10.1186/s40814-018-0266-8 (PMC5911950; doi:10.1186/s40814-018-0266-8)
Supplement: Supplementary file 1 — Table S1. Cost effectiveness analyses of self-management interventions in people with diabetes. (DOCX 28 kb) [file 40814_2018_266_MOESM1_ESM.docx]

Additional File 1.1

## Table 1: Cost effectiveness analyses of self-management interventions in people with diabetes

| **No.** | **Author** | **Title** | **Intervention Details** | **Pop./ Sample size** | **Country** | **Duration of study/ Time horizon** | **Outcome Measure** | **Costs** | **Findings** |
| --- | --- | --- | --- | --- | --- | --- | --- | --- | --- |
| 1 | Brown *et al. (2012)^1^* | Cost-Effectiveness analysis of a community health worker intervention for low-income Hispanic adults with diabetes | An ongoing community-based diabetes education and self-management programme that uses community partnerships and trained community health workers to reach participants.  Control: Standard Care | 30 | USA | 20 years; also report 5 and 10 years | QALYs were calculated in the Archimedes model. The model uses disutility weights for diabetes and illness. | Programme costs collected E.g. Staff costs, travel, volunteer time, participant time.  Project medical costs were modelled using the Archimedes model for healthcare expenditure. | ICERs ranging from $10,995 to $33,319 when compared with usual care. The programme was cost effective. |
| 2 | Shechter *et al. (2012)^2^* | Intervention Costs and Cost-Effectiveness of a Successful Telephonic Intervention to Promote Diabetes Control | Telephone intervention by health educators (behavioural counselling to promote change in lower-income, urban adults including medication adherence, healthy eating, increased physical activity) plus printed self-management education materials (up to 10 self-management phone calls)  Control: Printed self-management education materials (active control) | 526 | USA | 1 year | Mean decrease in HbA1C.  Proportion of participants achieving target A1C <7%. (goal) | Programme costs collected E.g. Staff costs; health educators making the call, supervision and training of health educators by nurse diabetes educator;  Phone charges;  Printing costs. | ICER of $491 per incremental percentage point of HbA1C improvement; ICER of $2,617 per person achieving the A1C goal.  The costs of a telephonic intervention for diabetes self-management support are moderate and commensurate to the modest associated improvement in glycemic control. |
| 3 | Ritzwoller *et al* (2011)^3^ | Intervention costs and cost-effectiveness for a multiple-risk-factor diabetes self-management trial for Latinas: economic analysis of ¡Viva-Bien! | A complex multi-risk-reduction lifest6yle intervention targeting low-income Latinas with type II diabetes.  Control: Standard Care | 280 | USA | 6 months | Percentage change in HbA1C.  Body Mass index (BMI) change | Programme costs collected E.g. Staff costs by job category ($/hr);  Printing costs;  Translation costs;  Participant costs collected; Out-of-pocket costs such as gym membership, exercise equipment, costs of dietary changes. | ICER of $5,076 per incremental change in BMI. This figure is similar for HbA1C.  Did not capture all participant cost-sharing measures. The analyses suggest that it may be possible to reduce costs and increase cost-effectiveness by reducing intervention intensity or by using phone/video response technologies. |
| 4 | Kaplan *et al* (1988)^4^ | The cost-utility of diet and exercise interventions in non-insulin dependent diabetes mellitus | Behaviour interventions: diet plus exercise programme.  Control: Education programme (standard care) | 76 | USA | 18 months | Well-years, using QWS (Quality of Well-being Scale) | Costs of medical supervision and tests (Physical exams, Blood tests, ECG evaluations). | Intervention group showed significant improvement. Authors calculate a cost-utility ratio of $10,870 per well-year; Very optimistic assumption that the effects would continue for more than 1 year, decreasing cost-utility to $5,435. |
| 5 | Handley *et al.* (2008)^5^ | Cost-Effectiveness of Automated Telephone Self-Management Support with Nurse Care Management Among Patients with Diabetes. | An automated telephone self-management (ATSM) support with nurse care management for adults with type 2 diabetes in San Francisco.  Control: Standard Care | 226 | USA | 1 year | QALYs; from SF-12 responses. (Brazier and Roberts) | Programme costs collected E.g. Staff costs; Development costs; Translation costs; Patient recruitment and follow-up time; maintenance costs; Call cost;  No transportation costs collected. | Estimate a gain of 0.012 QALY with ATSM relative to usual care. CUA values range from $65,167 per QALY gained when including all programme costs to $32,333 per QALY gained when including only on-going costs; falling within the range of accepted diabetes interventions. |
| 6 | Brownson *et al.* (2009) ^6^ | Cost-Effectiveness of Diabetes Self-management Programs in Community Primary Care Settings | Diabetes initiative (DI) a diabetes self-management programme in primary care and community settings in disadvantaged areas with notable health disparities.  Control: No Initiative (Standard Care) | 1273 | USA | Lifetime | QALYs using disutility weights for complications and mortality | Programme costs collected E.g. Staff costs (diabetes education classes; 1-to-1 self-management sessions);  Support groups; travel costs and telephone costs. | Intervention is estimated to reduce long-term complications. ICER of $39,563/QALY – below common bench mark of $50,000/QALY. |
| 7 | Bower *et al* (2^7^012) ^8^ | A cluster randomised controlled trial of the clinical and cost-effectiveness of a ‘whole systems’ model of self-management support for the management of long-term conditions in primary care: trial protocol | A self-management support for patients with diabetes, COPD and IBS, through an evidence based ‘whole systems’ model involving patient support, training for primary care teams and service re-organisation integrated into routine delivery within primary care. | 1728 | UK | 12 months | QALYs using EQ-5D | Participant questionnaire used collecting data on GP visits, practice nurse visits, community health and social care, inpatient and outpatient visits, out-of-pocket costs and costs of lost-productivity. | Study ongoing. |
| 8 | Ridell *et al* (2012) ^9^ | Cluster randomised controlled trial of a peer support program for people with diabetes: study protocol for the Australian peers for progress study. | Peer led, group based support; Diabetes self-management education and education manual.  Control: Standard care | 240 | Australia | 12 months | QALYs using EQ-5D,  Risk of CVD,  PHQ9,  HbA1C | Postal questionnaire used to collect data on GP visits, travel and waiting times, Insurance, visits to other health professionals, vaccinations, and inpatient stays. | Study ongoing. |
