# Supplementary material for: Developing and feasibility testing of data collection methods for an economic evaluation of a supported selfmanagement programme for adults with a learning disability and type 2 diabetes
Source: Pilot Feasibility Stud. 2018 Apr 23;4:80. doi: 10.1186/s40814-018-0266-8 (PMC5911950; doi:10.1186/s40814-018-0266-8)
Supplement: Supplementary file 2 — Table S2. Cost effectiveness analysis of interventions for people with learning disabilities. (DOCX 22 kb) [file 40814_2018_266_MOESM2_ESM.docx]

## Table 2 Cost effectiveness analysis of interventions for people with learning disabilities

| **No.** | **Author** | **Title** | **Intervention Details** | **Pop. / Sample**  **Size** | **Country** | **Duration of study/ Time horizon** | **Outcome Measure** | **Costs** | **Findings** |
| --- | --- | --- | --- | --- | --- | --- | --- | --- | --- |
| 1 | Beeken *et al* (2013) ^10^ | Piloting a manualised weight management programme (Shape Up-LD) for overweight and obese persons with mild-moderate learning disabilities: Study protocol for a pilot randomised controlled trial. | Shape Up-LD: a manualised programme for overweight and obese persons with mild-moderate learning disabilities. | 60 | UK | 6 months | QALYs using EQ-5D | General resource use for the programme and usual care group collected by way of participant interviews, using CSRI and costed at national rates using PSSRU, ONS and reference cost data. | Study ongoing |
| 2 | Strydom *et al* (2010) ^11^ | Service use and cost of mental disorder in older adults with intellectual disability | Not an intervention; a study of care costs and service usage.  OBJECTIVE: to report service use and costs of older people with ID – adults with intellectual disability (not Downs Syndrome) aged >60 years | 212 | UK | 1 year | Cost-of-Illness study | Supporters were interviewed using the CSRI, adapted for use in intellectual disabilities;  Service use included: accommodation, social care, nursing care, in-patient stays, primary and community care, aids and adaptations. | Gender, ID severity, hearing impairment, physical disorder and mental illness all have a significant relationship with costs.  The average weekly cost was £790 (£41,080 per year). Accommodation costs =71%. |
| 3 | Knobbe (1995) ^12^ | Benefit-Cost Analysis of Community Residential Versus Institutional Services for Adults With Severe Mental Retardation and Challenging Behaviours | Community residential services for adults with severe mental Adults with severe mental retardation and challenging behaviours who had been moved to a community setting  Comparator: Standard Care (institution) | 11 | USA | 2 years | Activity patterns, using Residential Lifestyle Inventory.  Social interaction patterns, using Social Network Lifestyle Analysis Form | Data collected through a combination of reviewing records and reports and interviewing participants and staff.  Public programme costs such as food, medical, accommodation, administration, staff costs (training, salaries and benefits), insurance, fuel and vehicle maintenance are included. | Community support is calculated to cost $111,123 per year, compared to institution costs of $117,277 per year. Overall, the community-based programme costs represented slight public savings over costs of the state institution. |
| 4 | Polder *et al* (2002) ^13^ | Healthcare costs of intellectual disability in the Netherlands: a cost-of-illness perspective | Top-down cost-of-illness study describing the healthcare costs of intellectual disability (ID) and other mental disorders in the context of other diseases; All healthcare costs in Netherlands in 1994 | N/A | Netherlands | 1 year | Cost-of-Illness study | Used data on healthcare costs for each sector from the Dutch Ministry of Health for 1994.  Key variables used: inpatient care and outpatient care and medications.  Excluded out-of-pocket costs, supporter costs and special education. | 9% of total disease specific costs can be ascribed to ID. Costs will inevitably increase because of the ageing population & increasing life expectancy among people with disabilities. |
| 5 | Robertson *et al* (2006) ^7^ | Longitudinal Analysis of the impact and Cost of Person-Centred Planning for People With Intellectual Disabilities in England | A person-centred plan developed for 93 people  Comparator: Standard Care, i.e. no plan | 93 | England  (4 localities) | 2 years | Activity in social networks – Data collected using proxy (informal/ formal). | Direct costs collected through discussion with consultant trainers; data collected on staff costs.  Indirect costs; Supporters interviewed and completed questionnaires based on a revised version of the CSRI. | Direct costs: $1,202 per participant. Indirect costs are negligible. Modest changes are found in areas of social networks; contact with family, friends and community based activities. |
| 6 | Tryer *et al* (2009) ^14^ | Neuroleptics in the treatment of aggressive challenging behaviour for people with intellectual disabilities: a randomised controlled trial | Haloperidol & risperidone in aggressive challenging behaviour in adults with ID. Health Technology Appraisal | 86 | England, Wales & Australia | 1 year | Modified Overt Aggression Scale  Quality of Life Questionnaire (QOL-Q) | Supporters were interviewed using the CSRI, collecting data on all specialised accommodation, health services and social care: | Placebo showed greatest reduction in aggression.  No significant important benefits of the drugs. |
