# Supplementary material for: Developing and feasibility testing of data collection methods for an economic evaluation of a supported selfmanagement programme for adults with a learning disability and type 2 diabetes
Source: Pilot Feasibility Stud. 2018 Apr 23;4:80. doi: 10.1186/s40814-018-0266-8 (PMC5911950; doi:10.1186/s40814-018-0266-8)
Supplement: Supplementary file 3 — Other Tables. (DOCX 16 kb) [file 40814_2018_266_MOESM3_ESM.docx]

**Additional File 3**

## Table 3: Medication Use from 61 participants –Phase I

| **Diabetes drug type** | **Obs (n=61)** | **% taking medication** |
| --- | --- | --- |
| Metformin | 48 | 79% |
| Metformin SR (e.g. Glucophage SR) | 8 | 13% |
| Gliclazide e.g. Diamicron | 20 | 33% |
| Glimepiride e.g. Amaryl | 12 | 20% |
| Sitagliptin e.g. Januvia | 7 | 11% |
| Vildagliptin e.g. Galvus | 1 | 2% |
| Ploglitazone e.g. Actos | 6 | 10% |
| Dapagliflozin e.g. Forxiga | 2 | 3% |
| Metformin in combination with Sitagliptin e.g. Janumet | 1 | 2% |

*BNF

## Table 4: What kind of accommodation do you live in at the moment? –Phase II

| **Baseline: Accommodation** | **Obs (%)** |  | **Follow-up: Accommodation** | **Obs (%)** |
| --- | --- | --- | --- | --- |
| Domestic housing | 47 (57.32) |  | Domestic housing | 39 (50.65) |
| Sheltered housing | 13 (15.85) |  | Sheltered housing | 13 (16.88) |
| Residential home | 5 (6.10) |  | Residential home | 4 (5.19) |
| Nursing home | 1 (1.22) |  | Nursing home | 1 (1.30) |
| Shared Supported house | 15 (18.29) |  | Shared Supported house | 20 (25.97) |
| Missing | 1 (1.22) |  | Missing | 0 (0.00) |
| Total | 82 (100.00) |  | Total | 77 (100.00) |

## Table 5: Diabetes Medications –Phase II

| **Drug Name** | **Recommended dose as per BNF, and clinician advice*** | **Price as per BNF** | **Cost for 4 months** |
| --- | --- | --- | --- |
| Metformin | 2000mg*per day | 500 mg, net price 28-tab pack = £1.58 | 27.46 |
| Metformin SR e.g. GlucophageSR) | 2000mg* per day | 1 g, 28-tab pack = £4.26 | 37.02 |
| Gliclazide e.g. Diamicron | 160mg once a day | 80 mg, net price 60-tab pack = £4.38 | 17.76 |
| Glimepiride e.g. Amaryl | 4mg daily | 4 mg, 30-tab pack = £14.24 | 57.75 |
| Sitagliptin e.g. Januvia | 100 mg once daily | 100 mg, 28-tab pack = £33.26 | 144.52 |
| Ploglitazone e.g. Actos | 30mg daily | 30 mg, 28-tab pack = £35.89 | 155.95 |
| Dapagliflozin e.g. Forxiga | 10mg daily | 10 mg, 28-tab pack = £36.59 | 158.99 |
| Exenatide e.g. Byetta | 10 micrograms twice daily | 10 microgram/dose prefilled pen (60 doses) = £68.24 | 276.75 |
| Exenatide ER e.g. Bydureon | 2 mg once weekly | 2-mg pen = £18.34 | 318.77 |
| Liraglutide e.g. Victoza | 1.2 mg once daily | 3 × 3-mL prefilled pens = £117.72. Each pen =15 doses of 1.2mg | 318.28 |

*(Dr Ramzi Ajjan) & BNF^15^

## Table 6: Medication Use (complete cases) –Phase II

| **Diabetes drug type** | **Treatment Arm** | **Obs SSM + UC: n=26**  **UC: n=29** | **% taking medication** |
| --- | --- | --- | --- |
| Metformin | SSM + UC | 14 | 53.85 |
|  | UC | 15 | 51.72 |
| Metformin SR (e.g. Glucophage SR) | SSM + UC | 3 | 11.54 |
|  | UC | 4 | 13.79 |
| Gliclazide e.g. Diamicron | SSM + UC | 5 | 19.23 |
|  | UC | 7 | 24.13 |
| Glimepiride e.g. Amaryl | SSM + UC | 2 | 7.69 |
|  | UC | 5 | 17.24 |
| Sitagliptin e.g. Januvia | SSM + UC | 0 | 0.00 |
|  | UC | 4 | 13.79 |
| Ploglitazone e.g. Actos | SSM + UC | 1 | 3.85 |
|  | UC | 5 | 17.24 |
| Dapagliflozin e.g. Forxiga | SSM + UC | 1 | 3.85 |
|  | UC | 0 | 0.00 |
| Exenatide e.g. Byetta | SSM + UC | 1 | 3.85 |
|  | UC | 0 | 0.00 |
| Exenatide ER e.g. Bydureon | SSM + UC | 1 | 3.85 |
|  | UC | 0 | 0.00 |
| Liraglutide e.g. Victoza | SSM + UC | 1 | 3.85 |
|  | UC | 0 | 0.00 |
